# Sepsis survivors present with higher values of cardiac index and velocity time integral in the emergency department

**DOI:** 10.1186/cc14217

**Published:** 2015-03-16

**Authors:** T Santos, M Schweller, C Gontijo-Coutinho, D Franci, P Nocera, T Guerra-Grangeia, J Matos-Souza, M Carvalho-Filho

**Affiliations:** 1 Unicamp, Campinas, Brazil

## Introduction

Myocardial depression is common among septic patients [1]. The aim of this study was to assess whether the values of cardiac index (CI) and velocity-time integral (VTI) calculated by echocardiography differ between survivors and nonsurvivors of sepsis.

## Methods

This was a prospective observational study. We included adult newly admitted septic patients, regardless of disease severity. Exclusion criteria were concomitant pregnancy or obstetric/gynecological sepsis and co-existing or terminal diseases that may limit life expectancy. At the moment of recruitment, additional exclusion criteria included: concomitant pulmonary embolism, trauma or acute ischemic coronary disease; pericardial tamponade; aortic valve disease; tachyarrhythmias and absence of adequate echocardiographic windows. Echocardiographic evaluations were made within the first 10 minutes of initiation of fluid therapy in the emergency room. All measurements and images were obtained with a 1.5 to 3.5 MHz phased array transducer using a standard cardiac preset. CI is the quotient of the cardiac output (CO) divided by the body surface area. The CO is the product of the stroke volume by the heart rate. Stroke volume is calculated as the product between aortic VTI (measured using pulsed-wave Doppler) and aortic cross-sectional area. The latter is calculated in the long axis parasternal window using the left ventricular outflow tract diameter measurement.

## Results

In 3 months, 58 patients were included. The average age was 46.6 years, and 36 were male. Overall mortality was 14%. We included 16 patients with sepsis syndrome, 27 patients with severe sepsis and 15 patients with septic shock. Severe sepsis patients presented with higher values of CI, when compared with sepsis syndrome and septic shock patients (3.46, 3.08 and 2.92 l/minute/m^2^, respectively, *P *= NS). The same occurred with VTI (19.27, 18.81 and 16.74 cm for severe sepsis, sepsis syndrome and septic shock, respectively; *P *= NS). Mean values of CI were lower in nonsurvivors of sepsis (2.51 vs. 3.35 L/minute/m^2^, *P *= 0.018). Mean values of VTI were also lower in nonsurvivors (14.83 vs. 19.01 cm, *P *= 0.022).

## Conclusion

In our study, nonsurvivors of sepsis presented with lower values of both CI and VTI in the emergency department. Therefore, CI and VTI may be good markers of sepsis severity and mortality in newly admitted patients. In addition, further studies are warranted to assess the role of CI and VTI as therapeutic targets.
